# Compressed Liquid (${{p}}$-${{\rho}}$-${{T}}$) Measurements of *trans*-1,2-Dichloroethene [R-1130(E)]

**DOI:** 10.1007/s10765-025-03526-4

**Published:** 2025-03-24

**Authors:** Tara J. Fortin, Stephanie L. Outcalt

**Affiliations:** National Institute of Standards and Technology, Material Measurement Laboratory, Applied Chemicals and Materials Division, 325 Broadway, Boulder, CO 80305-3328 USA

**Keywords:** Density, Hydrochloroolefins, Refrigerants, Vibrating-tube densimeter

## Abstract

Pressure-density-temperature ($p$-$\rho$-$T$) data for the refrigerant R-1130(E) (*trans*-1,2-dichloroethene) were measured in the compressed liquid phase using an automated vibrating-tube densimeter. Overall, the measurements covered temperatures from 270 K to 410 K and pressures from 0.5 MPa to 30 MPa. Relative combined, expanded (95% confidence level) uncertainties ranged from approximately 0.05% to 0.06%. Here, we present measurement results, along with comparisons to available literature data and to a generalized extended corresponding states model.

## Introduction

The refrigerant blend designated as R-514A [1] is being considered as a replacement for the hydrochlorofluorocarbon (HCFC) R-123 (2,2-dichloro-1,1,1-trifluoroethane) in centrifugal chillers and HVAC systems. R-514A is an azeotropic mixture consisting of 74.7% (mass/mass) of the hydrofluoroolefin (HFO) R-1336mzz(Z) (*cis*-1,1,1,4,4,4-hexafluoro-2-butene) and 25.3% (mass/mass) of the hydrochloroolefin (HCO) R-1130(E) (*trans*-1,2-dichloroethene) [2]. The development of accurate models to effectively analyze the performance of refrigerant blends such as R-514A requires reliable thermophysical property data. The thermophysical properties of R-1336mzz(Z) have been well-studied; consequently, a high-quality equation of state (EoS) written in terms of the Helmholtz energy exists for this fluid [3] and has been incorporated into REFPROP (version 10) [4]. In contrast, the availability of experimental data for R-1130(E), particularly over a wide range of temperatures and pressures, is far more limited. For example, there are three data sets reporting single-phase liquid density available in the literature for R-1130(E) [5–7], plus several sources reporting saturated densities [8–19]. Consequently, no reference EoS is currently available. Instead, there is a generalized extended corresponding states (ECS) model available for HFOs [20] that has previously been applied to R-1130(E) [6].

In this work, we report pressure-density-temperature ($p$-$\rho$-$T$) measurements in the compressed liquid phase for R-1130(E). Overall, these measurements covered temperatures from approximately 270 K to 410 K and pressures from approximately 0.5 MPa to 30 MPa. The measurements presented here were part of a larger project, the scope of which included comprehensive measurements of vapor–liquid equilibria (VLE) [21] and speed of sound [22]. These measurements are collectively being utilized in the development of a new Helmholtz-energy-explicit EoS.

## Materials and Methods

### Sample Materials

**Fig. 1:**
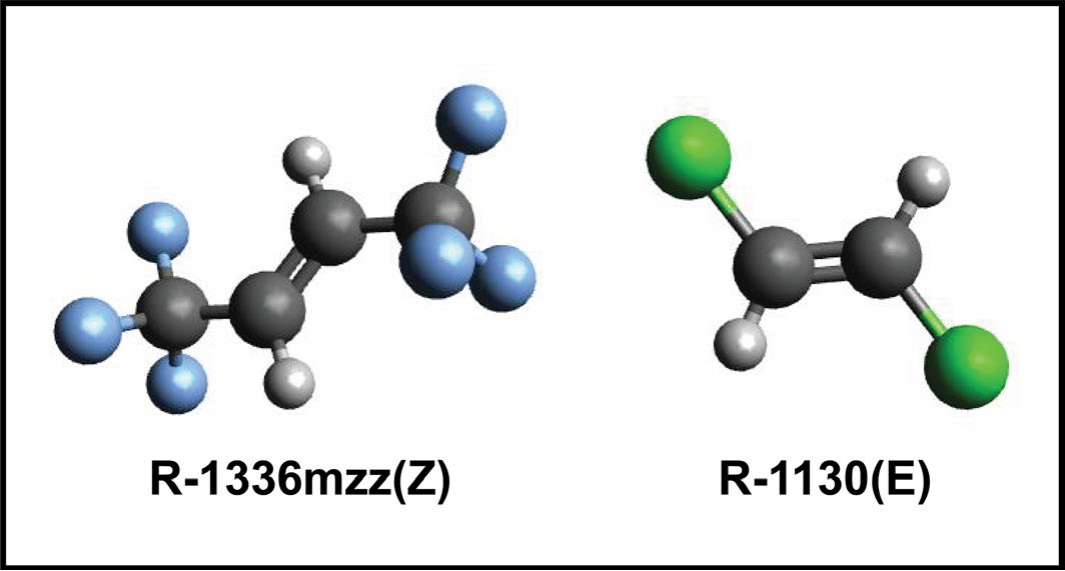
Molecular representations of refrigerants measured in this work. R-1336mzz(Z) was used as a calibration fluid. Carbon atoms are black, hydrogen atoms are gray, fluorine atoms are blue, and chlorine atoms are green. Diagrams were constructed using Avogadro [23]

Refrigerants measured in this work are listed in Table 1, along with the corresponding chemical formula, CAS number, supplier, and sample purity. Molecular representations constructed using Avogadro [23] are shown in Fig. 1. Both the calibration fluid, R-1336mzz(Z), and the primary measurement sample, R-1130(E), have purities of > 99%. The manufacturer’s stated purity for R-1336mzz(Z) was confirmed by gas-chromatography/quadrupole time-of-flight mass spectroscopy (GC/QToF-MS) measurements performed at NIST. For R-1130(E), purity was determined via a combination of GC/QToF-MS and nuclear magnetic resonance (NMR) spectroscopy measurements performed at NIST.

**Table 1:** Chemical information

Refrigerant	Chemical Name	Chemical Formula	CAS	Supplier^a^	Purity^b^
R-1336mzz(Z)	*cis*-1,1,1,4,4,4-hexafluoro-2-butene	C_4_H_2_F_6_	692-49-9	Chemours	0.9999
R-1130(E)	*trans*-1,2-dichloroethene	C_2_H_2_Cl_4_	156-60-5	Chemours	0.997

^a^In order to describe materials and experimental procedures adequately, it is occasionally necessary to identify commercial products by manufacturers’ names or labels. In no instance does such identification imply endorsement by the National Institute of Standards and Technology, nor does it imply that the particular product or equipment is necessarily the best available for the purpose

^b^Sample purity in mole fraction

Prior to measurements, each refrigerant was transferred from its manufacturer’s container to a clean stainless steel sample cylinder. The samples were then degassed to remove volatile impurities by freezing the sample in liquid nitrogen, evacuating the vapor space over the frozen sample, and then thawing the sample. This freeze–pump–thaw cycle was repeated a minimum of three times or until a negligible increase in pressure is observed during the evacuation step.

### Apparatus Description

A vibrating-tube densimeter (VTD) was utilized for the compressed liquid density measurements reported in this work. The instrument has been described in detail elsewhere [24] so only a brief description is provided here. At the heart of the apparatus is a commercial VTD with wetted parts made of Hastelloy C-276. Several modifications to the commercial instrument have been implemented, including the use of a custom-designed two-stage thermostat, which have improved overall temperature control, as well as the accuracy of both temperature and pressure measurements. As designed, the instrument operates over the temperature range from 270 K to 470 K and pressures up to 70 MPa. Temperature was measured with a standard platinum resistance thermometer (SPRT) that was calibrated using a series of fixed-point cells and was controlled with thin-film heaters; a circulating bath was also used for temperatures ≤ 300 K. Once thermal equilibrium was achieved, the instrument temperature remained stable to within ± 5 mK. The combined standard uncertainty in temperature was ≤ 15 mK. Pressure was measured with an oscillating quartz crystal pressure transducer that was calibrated using a NIST-traceable piston gauge and was controlled with a programmable syringe pump. The combined standard uncertainty in pressure was ≤ 5 kPa.

**Fig. 2:**
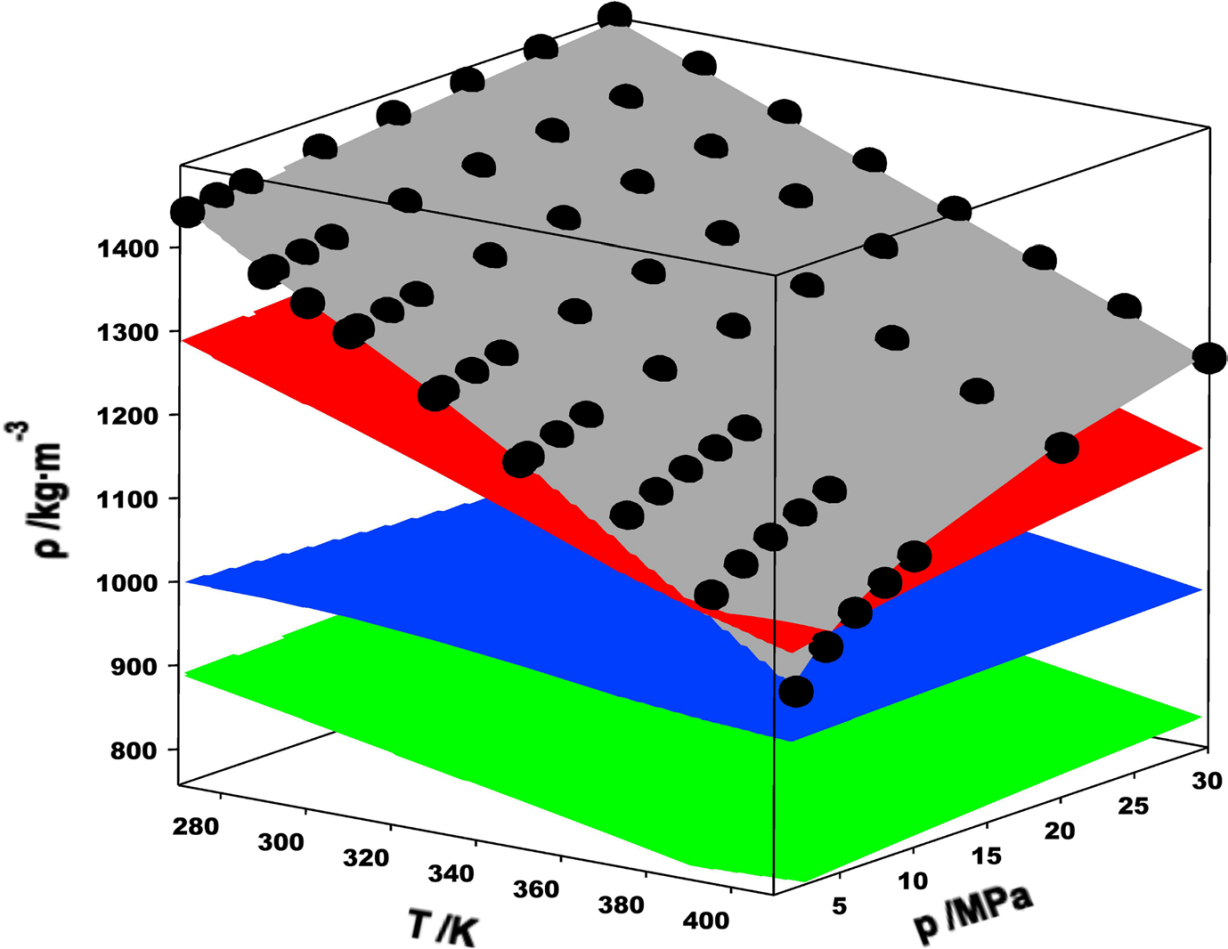
Selection of calibration fluids. The red surface represents R-1130(E) densities calculated with the ECS model of Teraishi et al. [6, 20], plotted vs temperature and pressure. The gray, blue, and green surfaces represent densities calculated with the EOS models included in REFPROP (Version 10.0) [4] for R-1336mzz(Z) [3], water [26], and toluene [25], respectively. Measured densities for R-1336mzz(Z) are also shown (●)

The fundamental principle of this measurement technique is that the period of oscillation ($\tau$) of a resonating hollow U-shaped tube can be related to the density ($\rho$) of the fluid that fills it. Mathematically, this can be expressed as

1 $$\rho =A{\tau }^{2}-B,$$

where $A$ and $B$ are apparatus-specific parameters that vary with both temperature and pressure and must be determined by calibration. Calibration involves first determining $\tau$ under vacuum (i.e., ${\tau }_{0}$) over the full temperature range of the instrument. Next, the tube is filled with a fluid for which the density is well-known and $\tau$ is measured over the full temperature and pressure range of interest. Ideally, the calibration fluid or fluids are chosen to cover the full range of expected densities for the sample fluid to be measured. In this work, we measured both toluene and R-1336mzz(Z) as potential calibration fluids but ultimately used just R-1336mzz(Z) for our instrument calibration. The motivation for this choice is illustrated in Fig. 2. In this figure, the black spheres represent measured R-1336mzz(Z) densities, the gray surface represents R-1336mzz(Z) reference densities [3], the red surface represents calculated densities for R-1130(E) using the ECS model of Teraishi et al. [6, 20], and the green surface represents toluene densities [25]. Also shown in Fig. 2 are water densities [26] (blue surface); water is commonly used as a calibration fluid for VTDs. Figure 2 clearly shows that both toluene and water densities are significantly lower than those for R-1130(E), while the R-1336mzz(Z) densities are very similar and include regions of overlap.

The choice of calibration fluid is not the only important consideration; the choice of calibration equation is also important. A more detailed discussion of this issue can be found in Outcalt [27]. In this work, we have used the physically-based model proposed by May et al. [28], which can be expressed in simplified form as

2 $$\rho = \frac{{\rho }_{\text{M}}/{\beta }_{1}}{1+{\beta }_{2}\cdot T+{\beta }_{3}\cdot p}\cdot \left({\left(\frac{\tau }{{\tau }_{0}}\right)}^{2}\cdot \left(1+{\beta }_{4}\cdot p\right)-1\right),$$

where ${\rho }_{\text{M}}$ is the density of the material from which the U-tube is constructed (Hastelloy), ${\beta }_{1}$, ${\beta }_{2}$, ${\beta }_{3}$, and ${\beta }_{4}$ are fitted parameters, $T$ is temperature, and $p$ is pressure. The final parameter, ${\tau }_{0}$, is obtained from a linear regression of the vacuum calibration data.

**Fig. 3:**
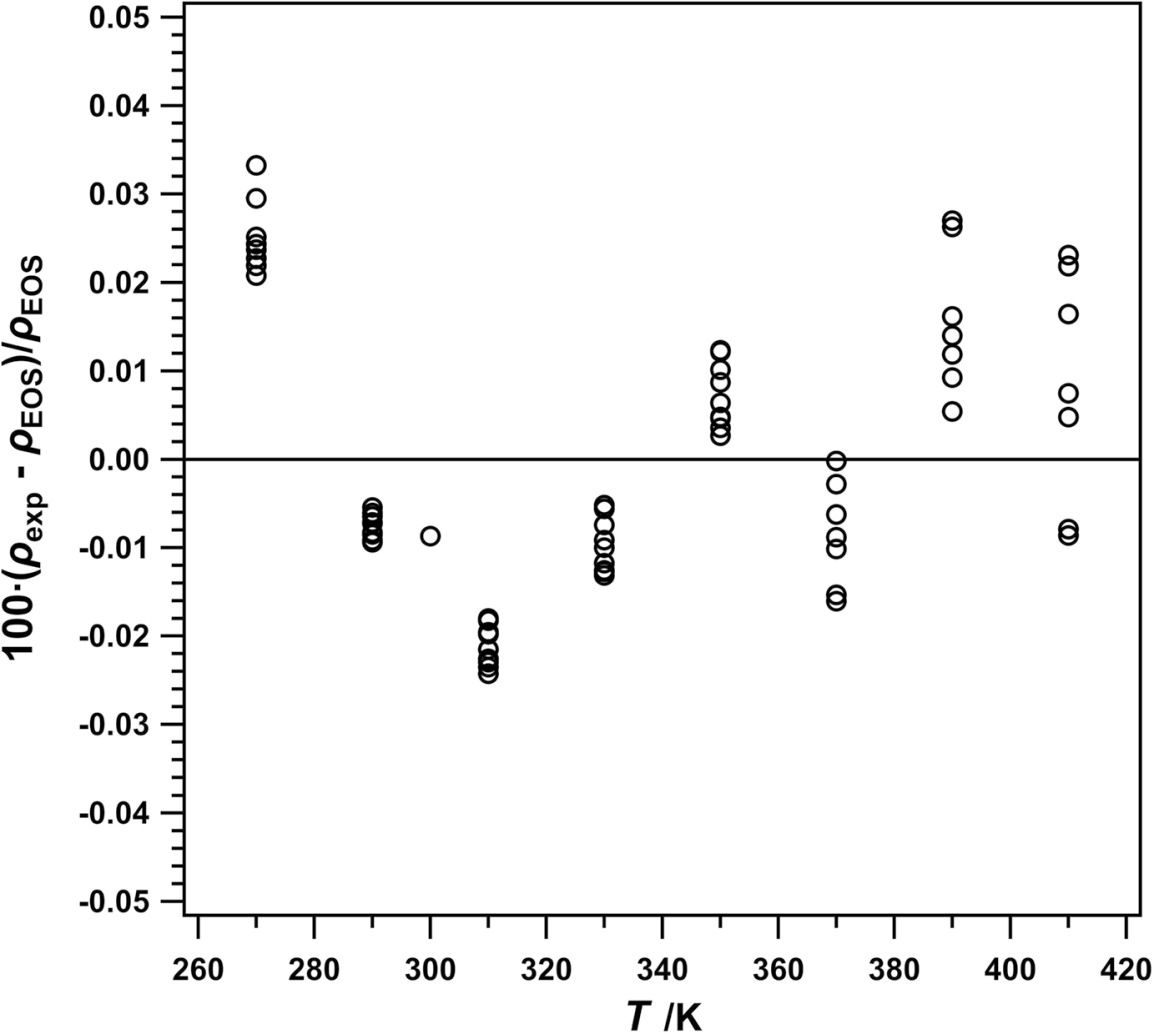
Relative deviations of measured ($p$, $\rho$, $T$) data for R-1336mzz(Z) from values calculated with the EOS [3] included in REFPROP (Version 10) [4], plotted as a function of temperature

The results of our calibration with R-1336mzz(Z) are shown in Fig. 3, plotted as percent deviation from the equation of state [3] included in REFPROP (version 10.0) [4] vs temperature. Here, the average absolute relative deviation (AARD) and the maximum relative deviation (MRD) were 0.01% and 0.03%, respectively. AARD was calculated as

3 $$\text{AARD}= 100\cdot \left\{\frac{1}{N}{\sum }_{i=0}^{N}\left|\frac{{\rho }_{\text{exp},i}-{\rho }_{\text{calc},i}}{{\rho }_{\text{calc},i}}\right|\right\},$$

and MRD as

4 $$\text{MRD}= \text{max}\left\{100\cdot \left|\frac{{\rho }_{\text{exp},i}-{\rho }_{\text{calc},i}}{{\rho }_{\text{calc},i}}\right|\right\},$$

where ${\rho }_{\text{exp},i}$ is the ${i}^{th}$ experimental density value, ${\rho }_{\text{calc},i}$ is the ${i}^{th}$ calculated density value, and $N$ is the total number of data points.

### Experimental Procedures

Prior to starting measurements, degassed liquid sample was loaded into the evacuated syringe pump and measurement cell. Once the VTD was filled with compressed liquid, measurements were made along isotherms starting from 270 K and increasing to 410 K in 20 K increments. For each isotherm, measurements were made from the highest to the lowest pressure, which in this work ranged from 30 MPa to an overall low of 0.5 MPa. Measurement conditions were chosen in an effort to ensure that the sample remained in the compressed liquid phase throughout. Once measurements have been completed for the full surface, additional measurements are typically made for at least two isotherms to check repeatability; poor repeatability can be an indication of sample decomposition. In this work, repeat measurements were performed for all eight isotherms.

## Results and Discussion

### Measurement Results

**Fig. 4:**
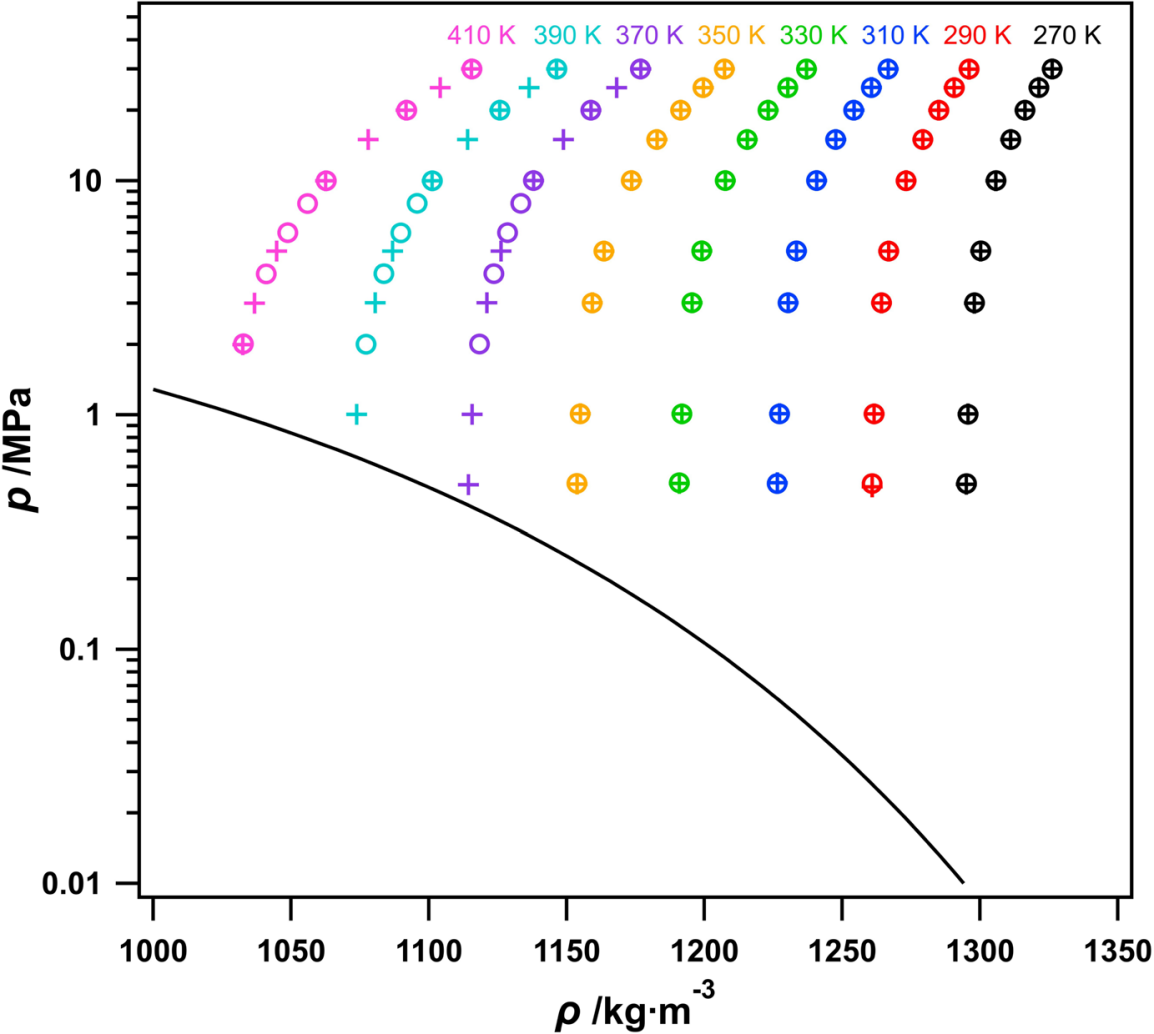
Measured ($p$, $\rho$, $T$) data for R-1130(E) plotted as pressure vs density. Here, ○ represent data for Run 1 and + represent data for Run 2. The liquid saturation line (—) was calculated using the ECS model of Teraishi et al. [6, 20]

The measured ($p$-$\rho$-$T$) state points for R-1130(E) are shown in Fig. 4, plotted as pressure vs density. In this figure, both sets of measurements, referred to as ‘Run 1’ and ‘Run 2’, are distinguished by different markers and isotherms are color-coded. Also shown in Fig. 4 is the liquid saturation line, which was calculated using the ECS model of Teraishi et al. [6, 20]. As intended, the sample appears to have remained in the compressed liquid state for all measured points. This was further verified by the absence of any instability in the measured $p$ and $\tau$, even at 370 K and 0.5 MPa.

The measured data shown in Fig. 4, are also presented in Table 2. Also included in Table 2 is the combined expanded uncertainty in density ($U(\rho )$), which was calculated as

5 $$U\left(\rho \right)={t}_{95}\left({df}_{\rho}\right)\cdot u\left(\rho \right),$$

where ${t}_{95}(df_{\rho})$ is the coverage factor taken from the *t*-distribution for $df_{\rho}$ degrees of freedom and a 95% level of confidence and $u(\rho )$ is the combined standard uncertainty in density. Corresponding ${t}_{95}(df_{\rho})$ values are included in Table 2 for clarity. The determination of $df_{\rho}$ and $u(\rho )$ are discussed in Sect. 3.2. It should be noted that reported values are averages of 10 replicate measurements at each ($T$, $p$) state point. Additionally, where there are nominally overlapping state points between Run 1 and Run 2, densities agree within 0.07 kg·m^−3^ on average.

**Table 2:** Measured Temperature ($T$), Pressure ($p$), and Density ($\rho$) Data for R-1130(E)^a^

$T$^b^	$p$^c^	$\rho$	${t}_{95}\left(df_{\rho}\right)$^d^	$U\left(\rho \right)$^e^	$T$^b^	$p$^c^	$\rho$	${t}_{95}\left(df_{\rho}\right)$^d^	$U\left(\rho \right)$^e^
/K	/MPa	/kg·m^−3^		/kg·m^−3^	/K	/MPa	/kg·m^−3^		/kg·m^−3^
Run 1					Run 2				
270.00	29.987	1326.19	2.36	0.66	270.00	29.985	1326.16	2.36	0.66
270.00	24.994	1321.34	2.36	0.66	270.00	25.009	1321.33	2.36	0.66
270.00	20.009	1316.35	2.36	0.66	270.00	20.004	1316.32	2.36	0.66
270.00	15.011	1311.18	2.36	0.66	270.00	15.005	1311.15	2.36	0.66
270.00	10.008	1305.82	2.36	0.66	270.00	10.003	1305.76	2.36	0.66
270.00	5.010	1300.28	2.36	0.66	270.00	5.001	1300.19	2.36	0.66
270.00	3.009	1297.99	2.36	0.66	270.00	2.997	1297.91	2.36	0.66
270.00	1.004	1295.68	2.36	0.66	270.00	1.009	1295.62	2.36	0.66
270.00	0.509	1295.10	2.36	0.66	270.00	0.505	1295.04	2.36	0.66
290.00	29.986	1296.07	2.36	0.65	290.00	29.972	1296.03	2.36	0.65
290.00	24.997	1290.65	2.36	0.65	290.00	24.995	1290.64	2.36	0.65
290.00	20.006	1285.05	2.36	0.65	290.00	19.996	1285.03	2.36	0.65
290.00	15.000	1279.22	2.36	0.65	290.00	15.009	1279.22	2.36	0.65
290.00	10.000	1273.15	2.36	0.65	290.00	10.010	1273.17	2.36	0.65
290.00	5.007	1266.85	2.36	0.65	290.00	5.004	1266.84	2.36	0.65
290.00	3.012	1264.23	2.36	0.65	290.00	3.007	1264.23	2.36	0.65
290.00	1.009	1261.55	2.36	0.65	290.00	1.010	1261.57	2.36	0.65
290.00	0.508	1260.88	2.36	0.65	290.00	0.492	1260.87	2.36	0.65
310.00	29.999	1266.59	2.36	0.65	310.00	30.014	1266.68	2.36	0.65
310.00	24.991	1260.52	2.36	0.65	310.00	25.009	1260.61	2.36	0.65
310.00	19.990	1254.19	2.36	0.65	310.00	20.003	1254.28	2.36	0.65
310.00	15.009	1247.61	2.36	0.65	310.00	15.008	1247.67	2.36	0.65
310.00	10.010	1240.68	2.36	0.65	310.00	10.009	1240.75	2.36	0.65
310.00	5.012	1233.38	2.36	0.65	310.00	5.009	1233.44	2.36	0.65
310.00	3.011	1230.34	2.36	0.65	310.00	3.010	1230.40	2.36	0.65
310.00	1.009	1227.23	2.36	0.65	310.00	1.012	1227.29	2.36	0.65
310.00	0.509	1226.43	2.36	0.65	310.00	0.512	1226.51	2.36	0.65
330.00	29.984	1237.04	2.36	0.64	330.00	29.980	1237.04	2.36	0.64
330.00	24.994	1230.24	2.36	0.64	330.00	25.005	1230.26	2.36	0.64
330.00	19.994	1223.06	2.36	0.64	330.00	20.014	1223.12	2.36	0.64
330.00	15.007	1215.52	2.36	0.64	330.00	15.007	1215.57	2.36	0.64
330.00	10.008	1207.53	2.36	0.64	330.00	10.008	1207.59	2.36	0.64
330.00	5.012	1199.04	2.36	0.64	330.00	5.012	1199.08	2.36	0.64
330.00	3.010	1195.47	2.36	0.64	330.00	3.010	1195.52	2.36	0.64
330.00	1.008	1191.80	2.36	0.64	330.00	1.009	1191.85	2.36	0.64
330.00	0.512	1190.86	2.36	0.64	330.00	0.509	1190.91	2.36	0.64
350.00	29.994	1207.22	2.36	0.64	350.00	29.987	1207.30	2.36	0.64
350.00	24.992	1199.51	2.36	0.64	350.00	24.995	1199.61	2.36	0.64
350.00	19.993	1191.37	2.36	0.64	350.00	19.990	1191.47	2.36	0.64
350.00	14.995	1182.72	2.36	0.64	350.00	14.994	1182.82	2.36	0.64
350.00	10.006	1173.48	2.36	0.64	350.00	10.014	1173.60	2.36	0.64
350.00	5.006	1163.50	2.36	0.64	350.00	5.008	1163.61	2.36	0.64
350.00	3.006	1159.27	2.36	0.64	350.00	3.010	1159.38	2.36	0.64
350.00	1.010	1154.89	2.36	0.64	350.00	1.010	1154.99	2.36	0.64
350.00	0.509	1153.76	2.36	0.64	350.00	0.507	1153.86	2.36	0.64
370.00	30.000	1176.81	2.36	0.63	370.00	30.000	1176.90	2.36	0.63
370.00	20.009	1158.82	2.36	0.63	370.00	24.996	1168.17	2.36	0.63
370.00	10.007	1138.00	2.36	0.63	370.00	19.991	1158.86	2.36	0.63
370.00	8.001	1133.36	2.36	0.63	370.00	14.994	1148.87	2.36	0.63
370.00	6.006	1128.58	2.36	0.63	370.00	10.007	1138.08	2.36	0.63
370.00	4.011	1123.60	2.36	0.63	370.00	5.009	1126.20	2.36	0.63
370.00	2.007	1118.37	2.36	0.63	370.00	3.008	1121.09	2.36	0.63
390.00	29.993	1146.46	2.36	0.62	370.00	1.004	1115.75	2.36	0.63
390.00	19.997	1125.80	2.36	0.62	370.00	0.503	1114.36	2.36	0.63
390.00	10.005	1101.29	2.36	0.62	390.00	29.990	1146.34	2.36	0.62
390.00	8.003	1095.74	2.36	0.62	390.00	24.996	1136.41	2.36	0.62
390.00	5.992	1089.83	2.36	0.62	390.00	19.992	1125.70	2.36	0.62
390.00	4.004	1083.73	2.36	0.62	390.00	14.994	1114.04	2.36	0.62
390.00	2.004	1077.24	2.36	0.62	390.00	9.998	1101.19	2.36	0.62
410.00	29.996	1115.66	2.36	0.62	390.00	5.006	1086.83	2.36	0.62
410.00	19.991	1091.97	2.36	0.62	390.00	3.008	1080.54	2.36	0.62
410.00	9.993	1062.83	2.36	0.62	390.00	1.006	1073.85	2.36	0.62
410.00	7.995	1056.04	2.36	0.62	410.00	30.003	1115.46	2.36	0.62
410.00	5.995	1048.81	2.36	0.62	410.00	24.981	1104.09	2.36	0.62
410.00	3.996	1041.05	2.36	0.62	410.00	19.998	1091.73	2.36	0.62
410.00	2.010	1032.75	2.36	0.62	410.00	15.001	1078.04	2.36	0.62
					410.00	9.997	1062.58	2.36	0.62
					410.00	5.002	1044.77	2.36	0.62
					410.00	3.000	1036.75	2.36	0.62
					410.00	1.996	1032.47	2.36	0.62

^a^Reported values are averages of 10 replicate measurements at each ($T$, $p$) state point

^b^Standard uncertainty in temperature is ~ 15 mK

^c^Standard uncertainty in pressure is ~ 5 kPa

^d^Coverage factor from the *t*-distribution for $df_{\rho}$ degrees of freedom and a 95% confidence level

^e^Combined, expanded (95% confidence level) uncertainty in density

### Measurement Uncertainty

The main sources of uncertainty for the density data collected with the VTD are the uncertainties associated with the temperature and pressure measurements and the uncertainty associated with the instrument calibration. Given that the measurement sample in this work was not as pure as we would hope for with a single-component fluid, uncertainties associated with composition have also been included. Therefore, the combined standard uncertainty in the reported R-1130(E) densities, $u\left(\rho \right)$, can be expressed as

6 $$u\left(\rho \right)= \sqrt{{u\left(T\right)}^{2}+{u\left(p\right)}^{2}+{u\left(cal\right)}^{2}+{u\left(x\right)}^{2}},$$

where the combined standard uncertainties for temperature ($u(T)$), pressure ($u(p)$), instrument calibration ($u(cal)$), and composition ($u(x)$), are all in units of kg·m^−3^. In this work, $u(T)$ included contributions from the uncertainties in the SPRT, the multimeter used to read the SPRT, the temperature calibration, temperature stability, and temperature gradients. Estimates for the individual contributions were combined using root sum of squares (RSS) to yield a value of 15 mK, which corresponds to $u(T)$ = 0.04 kg·m^−3^. Similarly, $u(p)$ included contributions from the uncertainties associated with the transducer, the pressure calibration, the applied zero-pressure correction, and pressure stability. When combined using RSS, the resulting uncertainty was 5 kPa, which corresponds to $u(p)$ = 0.02 kg·m^−3^. The calibration uncertainty, $u(cal)$, included contributions from the EOS for R-1336mzz(Z), the vacuum calibration, and the fit to the calibration equation; when combined using RSS, $u(cal)$ was approximately 0.2 kg·m^−3^. Finally, $u(x)$ was approximately 0.17 kg·m^−3^; this estimate was derived from sensitivity tests using REFPROP (version 10) [4] where we varied the relative concentrations of the three primary impurities identified during the compositional analysis (R-1336mzz(Z), 1,2-epoxybutane, and water) and compared the resulting densities to predicted values for a 100% pure fluid.

As was discussed in Sect. 3.1, the combined expanded uncertainty in density, $U(\rho )$, was calculated according to Eq. 5 using the $u\left(\rho \right)$ values calculated with Eq. 6 and the coverage factor, ${t}_{95}({df}_{\rho})$. Determination of the coverage factor first requires an estimation of the corresponding degrees of freedom, ${df}_{\rho}$, which was calculated using the Welch-Satterthwaite approximation [29]:

7 $${df}_{\rho}= \frac{{u\left(\rho \right)}^{4}}{\left(\frac{{u\left(T\right)}^{4}}{{df}_{T}}+\frac{{u\left(p\right)}^{4}}{{df}_{p}}+\frac{{u\left(cal\right)}^{4}}{{df}_{cal}}+\frac{{u\left(x\right)}^{4}}{{df}_{x}}\right)},$$

where ${df}_{T}$, ${df}_{p}$, ${df}_{cal}$, and ${df}_{x}$ are the corresponding degrees of freedom for $u(T)$, $u(p)$, $u(cal)$, and $u(x)$, respectively. In this work, ${t}_{95}(df_{\rho})$ was 2.36 and $U(\rho )$ ranged from 0.62 kg·m^−3^ to 0.66 kg·m^−3^, corresponding to relative combined expanded uncertainties of approximately 0.05% to 0.06%.

### Data Comparisons

As was previously mentioned, the comprehensive thermophysical property measurements from this project are being used to develop a Helmholtz energy empirical multiparameter EoS for R-1130(E); that work is currently in progress. Meanwhile, here we have compared our ($p$-$\rho$-$T$) data with the existing ECS model of Teraishi et al. [20]; this is a purely predictive model that uses universal parameters that are generalized to HFOs. The model reportedly represents saturated liquid densities to within 3% for well-studied HFOs and is believed to be perform similarly for other HFOs and hydrochlorofluoroolefins (HCFOs) if there are reliable critical temperature, critical density, and acentric factor data available [20]. For R-1130(E), we are using the model as implemented by Tanaka et al. [6].

**Fig. 5:**
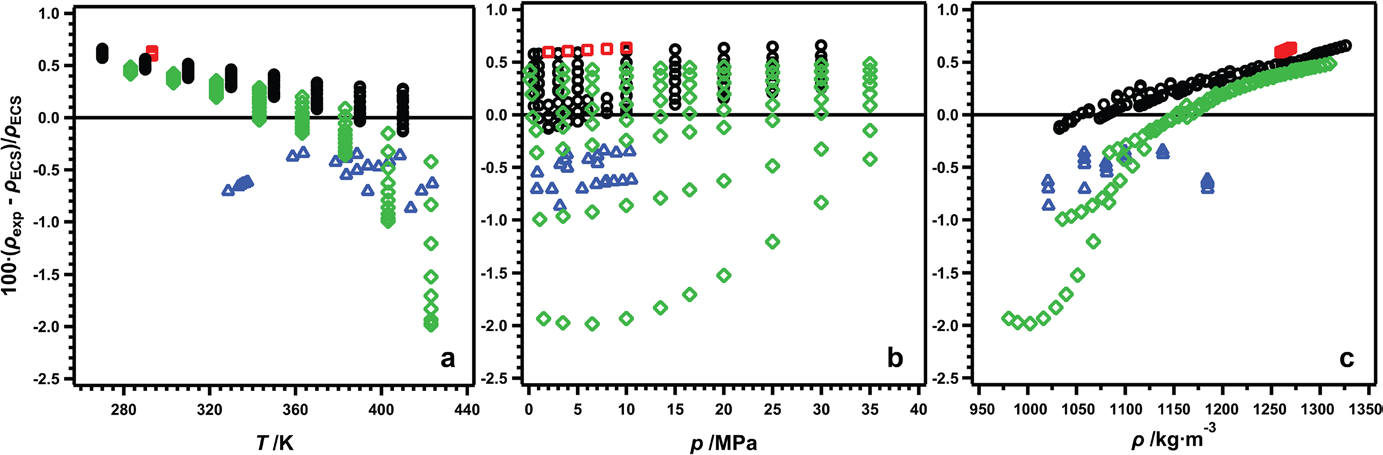
Relative deviations of experimental ($p$, $\rho$, $T$) data for R-1130(E) from values calculated with the ECS model of Teraishi et al. [6, 20], plotted as a function of (a) temperature, (b) pressure, and (c) density. For all graphs: ○, this work; , Hahn et al. [5]; and , Tanaka et al. [6]; and , Lombardo et al. [7]

Figure 5 shows the results of these comparisons plotted as percent deviation vs temperature (Fig. 5a), pressure (Fig. 5b), and density (Fig. 5c). Results from this work are shown as black circles. Also shown in Fig. 5 are comparisons with available literature data. As was mentioned previously, the available literature data for R-1130(E) are limited. Here we compare with the three data sets for which there are single-phase liquid densities available: Hahn et al. [5] (red squares), Tanaka et al. [6] (blue triangles), and Lombardo et al. [7] (green diamonds). The AARD and MRD have been calculated relative to the ECS model according to Eqs. 3 and 4, respectively, to aid in these comparisons; the results are listed in Table 3. The results from this work show the best agreement with the ECS model with an AARD of 0.34%, while the results of Hahn et al. [5] show the worst agreement with an AARD of 0.62%. The remaining two data sets fall in between with AARDs of 0.47% for Lombardo et al. [7] and 0.55% for Tanaka et al. [6]. With the exception of a handful of data points, none of the experimental data agree with the ECS model within the maximum reported uncertainties of 0.06% for this work, Hahn et al. [5], and Lombardo et al. [7], and 0.11% for Tanaka et al. [6]. However, even the largest deviations of 1.98% are well within the model’s reported 3% uncertainty for saturated liquid densities.

**Table 3:** Relative deviations of experimental data from values calculated with the ECS Model [6, 20]

Data source	AARD^a^	MRD^b^
This work	0.34	0.66
Hahn et al. [5]	0.62	0.64
Tanaka et al. [6]	0.55	0.87
Lombardo et al. [7]	0.47	1.98

^a^Average absolute relative deviation calculated according to Eq. 3

^b^Maximum relative deviation calculated according to Eq. 4

Although Fig. 5 shows regions of reasonable agreement between data sets, there are clearly also regions where significant discrepancies exist, particularly at the highest temperatures and the lowest pressures and densities. The question is whether or not the observed discrepancies can be explained by experimental differences between the data sets. In all instances, the reported sample purities are less than 99.9% making composition a possible contributing factor. However, without additional information regarding the impurities present in the other samples it is difficult to draw any conclusions regarding the possible extent of this contribution. Of the four data sets discussed here, all but that of Tanaka et al. [6] were measured using a vibrating-tube densimeter. Given the importance of calibration with these instruments, the possibility of calibration differences being reflected in the results needs to be considered.

As was discussed in Sect. 2.2, the VTD used in this work was calibrated with R-1336mzz(Z) and the equation of May et al. [28]. Hahn et al. [5] calibrated with benzene and carbon tetrachloride; no details regarding the equation used were provided. Lombardo et al. [7] calibrated with water and a calibration equation of their own. Both benzene and water have densities significantly lower than R-1130(E), as well as the R-1336mzz(Z) used in this work, while carbon tetrachloride has densities that are significantly higher. While we did not measure benzene, water, or carbon tetrachloride in this work, we did measure toluene. As shown in Fig. 2, toluene has densities that are lower than those of water; the surface for benzene is not shown in Fig. 2 but it has densities that are similar to, though slightly higher than, those of toluene.

**Fig. 6:**
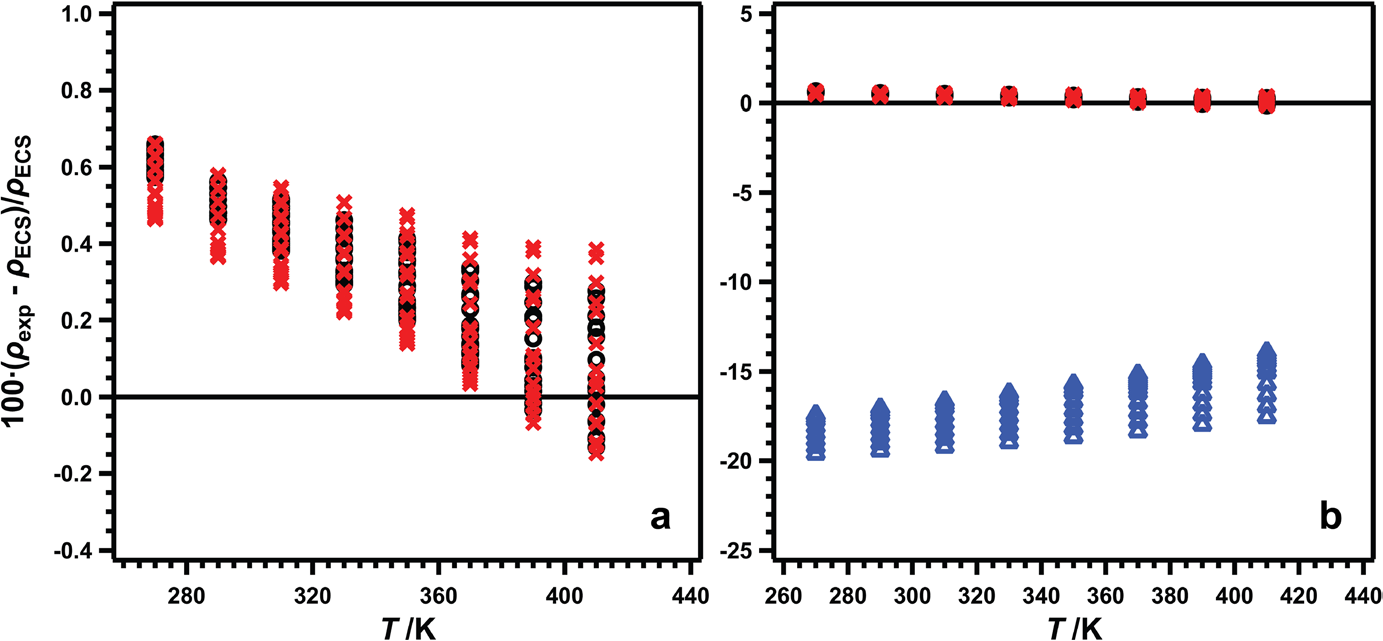
Testing the impact of calibration choices on density results. Results are plotted as relative deviations of experimental (${p}$-${\rho}$-${T}$) data for R-1130(E) from values calculated with the ECS model of Teraishi et al. [6, 20] vs temperature. The calibration fluid impact is shown in (a), while the calibration equation impact is shown in (b). For all graphs: ○, R-1336mzz(Z) and May et al. [28] equation; , toluene and May et al. [28] equation; , toluene and 9-term polynomial.

To test the influence of the choice of calibration fluid, we processed our data using toluene instead of R-1336mzz(Z), while still using the equation of May et al. [28]. These results are shown in Fig. 6a where the black circles are the previously reported results, and the red x’s are the recalculated results using toluene as the calibration fluid. Here, using toluene as the calibration fluid did not have a significant impact on the results, despite having significantly lower densities. The AARD and MRD for this case are 0.32% and 0.66%, respectively, compared to 0.34% and 0.66% for R-1336mzz(Z) (Table 3). In past work, both a 13-term [24] and a 9-term [27] polynomial have been used as the calibration equation; here we tested the influence of the calibration equation by processing our data using toluene and a 9-term polynomial. These results are shown in Fig. 6b as blue triangles; for comparison, the results from Fig. 6a are also shown. In contrast to the first test (Fig. 6a), changing the calibration equation had a significant impact on the resulting R-1130(E) densities. The resulting AARD and MRD are 17.0% and 19.6%, respectively, approximately an order of magnitude larger than the worst of the previously observed deviations. Such large deviations could indicate an issue with overfitting and/or problematic extrapolation behavior with the 9-term polynomial. While these tests do not provide a definitive explanation for the observed differences between data sets (Fig. 5), it does highlight the potentially dramatic impact that the choice of calibration fluid and/or equation can have on the results.

## Conclusions

The ($p$-$\rho$-$T$) measurement results for R-1130(E) have been reported in this work. The measurements covered temperatures from 270 to 410 K and pressures from 0.5 MPa to 30 MPa and were conducted using an automated vibrating-tube densimeter. At these temperatures and pressures, the sample remained in the compressed liquid phase throughout, and the measured densities ranged from approximately 1032 kg·m^−3^ to 1326 kg·m^−3^. Data comparisons show regions of reasonable agreement with available literature data but also show regions where significant differences are observed, particularly at higher temperatures and lower pressures and densities. Comparisons with the existing ECS model of Teraishi et al. [6, 20] showed an AARD of 0.34% for this work; in comparison, AARDs for available literature data ranged from 0.47% to 0.62%. With a few exceptions, the observed deviations exceeded reported experimental uncertainties but were within the uncertainty of the model. The density data presented here expand upon the limited existing data sets for R-1130(E) and are therefore invaluable to ongoing modeling efforts aimed at the development of a new Helmholtz-energy-explicit EoS for this fluid.

## Data Availability

The (*p*-*ρ*-*T*) data presented in Table 2 are available for download as tab-delimited text files at 10.18434/mds2-3429.
